# Lysosomal Functions in Glia Associated with Neurodegeneration

**DOI:** 10.3390/biom11030400

**Published:** 2021-03-09

**Authors:** Conlan Kreher, Jacob Favret, Malabika Maulik, Daesung Shin

**Affiliations:** 1 Department of Biotechnical and Clinical Laboratory Sciences, Jacobs School of Medicine and Biomedical Sciences, University at Buffalo-SUNY, Buffalo, NY 14214, USA; conlankr@buffalo.edu (C.K.); jacobfav@buffalo.edu (J.F.); malabika@buffalo.edu (M.M.); 2 Hunter James Kelly Research Institute, Jacobs School of Medicine and Biomedical Sciences, University at Buffalo-SUNY, Buffalo, NY 14203, USA

**Keywords:** lysosomes, glia, autophagy, synapse, astrocytes, oligodendrocytes, microglia, neurodegenerative diseases

## Abstract

Lysosomes are cellular organelles that contain various acidic digestive enzymes. Despite their small size, they have multiple functions. Lysosomes remove or recycle unnecessary cell parts. They repair damaged cellular membranes by exocytosis. Lysosomes also sense cellular energy status and transmit signals to the nucleus. Glial cells are non-neuronal cells in the nervous system and have an active role in homeostatic support for neurons. In response to dynamic cues, glia use lysosomal pathways for the secretion and uptake of regulatory molecules, which affect the physiology of neighboring neurons. Therefore, functional aberration of glial lysosomes can trigger neuronal degeneration. Here, we review lysosomal functions in oligodendrocytes, astrocytes, and microglia, with emphasis on neurodegeneration.

## 1. Introduction

Lysosomes are membrane-bound organelles that serve as the primary site of macromolecular catabolism. These dynamic organelles are functionally and morphologically heterogeneous, ranging in size from 200 to 1000 nm in diameter and interacting with multiple critical cellular pathways, including metabolic pathways, secretory pathways, and even signal transduction (reviewed in [1]). A critical property of lysosomes is their acidic pH, ranging between 4.5 and 5.5, which is maintained by the vacuolar H^+^ ATPase (v-ATPase) [2]. This acidic environment enables the multitude of acidic hydrolases found in the lysosomes to function, degrading a wide array of macromolecules into amino acids, monosaccharides, and free fatty acids [3]. 

Extracellular materials are internalized by endocytosis into the cells and transported into a lysosome to be digested by acid hydrolases. Endocytosis includes pinocytosis and phagocytosis [4,5]. Phagocytosis is typically viewed as a major mechanism of the innate immune system, due to the formation of the phagosome initiated by specialized cell surface receptors such as the Fc, IgG, mannose 6-phosphate and complement receptors [6]. Endocytosis can be initiated in a variety of ways, from clathrin- and caveolin-dependent and -independent endocytosis, to cytoskeletal initiated pinocytosis [7]. Apart from the intake and catabolism of extracellular materials, cells also require machinery to degrade damaged organelles, unused proteins, and other intracellular waste products. This process of self-recycling is known as autophagy and can be divided into three major forms; macroautophagy, microautophagy, and chaperone-mediated autophagy (CMA) [8]. CMA involves the transfer of cytoplasmic proteins attached to chaperones to the lysosome via receptor-mediated trafficking [9]. Microautophagy is mediated by direct engulfment of cytosolic materials into lysosomes and is involved in nutrient recycling along with macroautophagy [10]. Macroautophagy, henceforth referred to as autophagy, is the primary method the cell uses to degrade damaged organelles or unused proteins, particularly proteins with long half-lives, as the proteasome is the preferred degrative pathway for short-lived or tightly regulated proteins [11]. Autophagy is critical for the cellular response to stress, from starvation, oxidative stress, mitochondrial damage, endoplasmic reticulum (ER) stress, or pathogen invasion [8]. Lysosomes also participate in exocytosis, which allow them to fuse with the plasma membrane, thereby releasing hydrolytic compartments to the extracellular space [12]. Lysosomal exocytosis can function to signal the immune system, release hydrolases that aid in tissue remodeling, propagate cell signaling, dispose of indigestible cellular aggregates, and repair the damaged plasma membrane [13,14,15,16].

Energy homeostasis is a critical and evolutionarily conserved function of the lysosome, with homologous proteins existing in prokaryotes, fungi, and eukaryotes [17]. Two main pathways modulate nutrient and energy homeostasis: the AMP-activated protein kinase (AMPK) and the mechanistic target of rapamycin complex 1 (mTORC1) pathways. AMPK is a serine/threonine kinase that regulates ATP production and, in part exerts its effects via inhibition of mTORC1 [18]. mTORC1 serves as a key regulator of a wide range of cellular homeostasis: protein/lipid synthesis and energy metabolism. Its activity is modulated by many factors such as growth factors, amino acids, stress, energy status, and oxygen [19]. For example, mTORC1 is activated after sensing the upregulated level of arginine in the lysosome via Solute Carrier Family 38 Member 9 (SLC38A9) [20,21,22].

About 90% of all cells in the human brain are non-neuronal glial cells. Although glial cells are electrically inert, they are present as diverse forms of cell type, playing a critical role in the regulation of brain homeostasis. Astrocytes link the vasculature and neurons transporting multiple metabolites, including glucose, which is the main source of energy consumed by neurons. Astrocytes have also an active role in the recycling of neurotransmitters such as glutamate. Oligodendrocytes generate myelin structure to wrap around neuronal axons for the saltatory conduction of action potentials. Oligodendrocytes also provide several local trophic metabolites to long axonal tracts of neurons [23]. As immune cells, microglia surveil the health of the brain function and cleanse debris to maintain central homeostasis. Microglia become reactive and concentrate at the site of brain injury to phagocytize damaged cells. Furthermore, they remove unnecessary synaptic connections during brain development. There is emerging evidence pointing towards glial cells being critical determinants in multiple aspects of brain development [24], and subsequently, in neuronal degeneration [25,26]. Many neurodegenerative diseases such as Parkinson’s disease (PD), Alzheimer’s disease (AD), Huntington’s disease, and frontotemporal dementia present with protein aggregations that appear to overwhelm the autophagosome-lysosome pathway [27,28,29]. The disruption of the autophagic pathway can disrupt the delicately balanced homeostasis found in the interwoven network of cells in the nervous system. Here, we review how the lysosomes in each glial cell type regulate the homeostasis of the central nervous system (CNS) and discuss how dysfunctional glial lysosomes result in neurodegenerative diseases.

## 2. Lysosomal Function in Oligodendrocytes

Oligodendrocytes synthesize the myelin sheath, a specialized membranous structure insulating neuronal axons [30]. Myelin sheath is essential for the conductance of action potential and for providing metabolic support to the long axonal tracts of neurons [31]. Oligodendrocytes are generated from oligodendrocyte precursor cells (OPCs), some of which differentiate during brain development while others continue to proliferate into adulthood becoming adult OPCs [32]. The adult OPCs can differentiate into myelinating oligodendrocytes forming new myelin structure, in response to various internal and external cues for myelin plasticity. Myelin is comprised of trans- or peripheral-membrane proteins such as myelin basic protein (MBP), proteolipid protein (PLP), and myelin-associated glycoprotein (MAG) [33]. The expression and proper localization of these proteins are crucial for the myelination process, which is carried out by oligodendrocytes and Schwann cells in the central and peripheral nervous systems, respectively. The main functions of these proteins include compacting myelin structure, conducting axonal signaling, and maintaining the axon–myelin associations [33]. Myelin synthesis is a very intricate and tightly regulated process. The key elements of myelination comprise the synthesis, storage, and transportation of myelin proteins [34]. Since lysosomes are important machinery for protein secretion and sorting, it is imperative to understand how the lysosomal pathway modulates the recycling of myelin proteins in ensuring the proper functioning of the oligodendrocytes.

### 2.1. Oligodendrocyte Exocytosis

**Figure 1:**
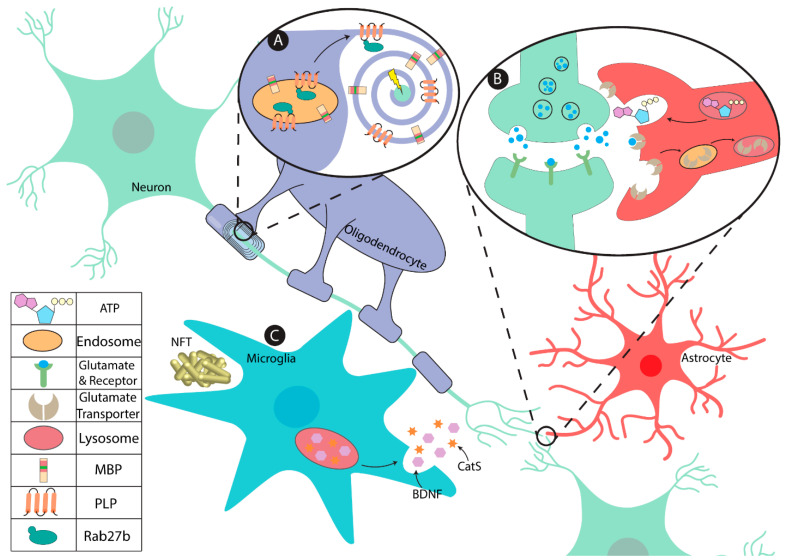
Lysosomal function in the major glial cell types in the central nervous system (CNS). (**A**) Lysosomes are integral in establishing and maintaining proper myelin integrity in oligodendrocytes. The lysosomal/endosomes in oligodendrocytes sort and transport myelin proteins such as PLP, which is co-trafficked by Rab27b, and MBP to the myelin sheath for myelin turnover and plasticity in an activity dependent manner. (**B**) Astrocytic lysosomes are key modulators of the extracellular environment of the synaptic cleft. Their lysosomes secrete ATP, modulating synaptic strength. Furthermore, the recycling of glutamate is mediated via endocytosis from the peri-synaptic membrane, thus influencing synaptic glutamate availability. (**C**) Microglia sequester neurodegenerative substances that accumulate in proteinopathies such as neurofibrillary tangles (NFT) associated with Alzheimer’s disease. Microglial lysosomes are implicit in releasing multiple factors including brain-derived neurotrophic factor (BDNF) and Cathepsin S (CatS) that aid in CNS development, memory formation, and remodeling of the extracellular matrix and synaptic architecture.

Lysosomes, in addition to their degradative role, respond to external stimuli resulting in exocytosis that is mediated by a group of SNARE proteins [35]. Lysosomal exocytosis has been reported in the CNS as a mode of secretory protein release from oligodendrocytes for myelin plasticity. The targeting process of myelin proteins is controlled by bidirectional communications between neurons and oligodendrocytes. PLP is expressed in the rough ER of oligodendrocytes that undergoes vesicular transport to the Golgi and plasma membrane, finally forming the myelin sheath with neuronal signals. Signals from the neuron can trigger the release of PLP from late endosomes/lysosomes (LE/Lys) stores to the plasma membrane during myelination [36]. The transport of PLP from recycling endosomes (REs) to the plasma membrane as well as exocytosis of lysosome-related organelles delivering cargo to the myelin sheath are mediated by pathways involving SNARE proteins such as Vesicle-associated Membrane Protein 3 (VAMP3) and VAMP7 [37]. Therefore, mutant mice with aberrant lysosomal exocytosis due to defects in VAMP7 sorting had mild demyelination featured by the diminished levels of myelin proteins, including PLP [37]. Among the members of the Rab family which are involved in lysosomal exocytosis, Rab27b was found to be colocalized with PLP in oligodendrocytes LE/Lys (Figure 1A). Rab27b knockdown in cell culture models significantly reduced lysosomal exocytosis and reduced PLP expression on the surface of oligodendrocytes [38]. It was also shown that in a cathepsin D knockout mouse model which is a lysosomal proteinase enzyme, both PLP and MBP levels were significantly reduced with marked degeneration of myelin sheath [39]. Furthermore, the maturation of myelin was significantly delayed in the cathepsin D knockout mice, due to the defective targeting of PLP to the plasma membrane. The impaired targeting of PLP to the myelin membranes might be caused by its abnormal interaction with cathepsin D and VAMP7 in late endosomes, resulting in the delayed myelin formation [40].

In the peripheral nervous system (PNS), myelin sheath is generated by Schwann cells [41]. The activity of the myelinating Schwann cells is regulated by inputs from the axons and the extracellular matrix. In an event of injury, Schwann cells dedifferentiate back to a proliferative state to support the neuronal survival [42]. Lysosomal exocytosis in Schwann cells also contributes to the myelination process in the PNS [43]. For example, Rab27a GTPase is essential for secretory Ca^2+^ lysosome trafficking in Schwann cells and thus for myelination. The myelin protein P0 was colocalized with Rab27a in the LE/Lys of Schwann cells. The Ca^2+^ induced lysosomal exocytosis in Schwann cells was significantly reduced in the Rab27a-knockdown Schwann cells. Finally, in the Rab27a knockout mouse model, after a sciatic nerve injury the remyelination of the injured axon was impaired [43], indicating the importance of secretory lysosomes in the peripheral nerve regeneration by Schwann cells.

### 2.2. Oligodendrocyte Phagocytosis/Endocytosis

Research suggests that trafficking of myelin proteins through LE/Lys might play an important role in axonal signal-mediated myelin biogenesis [36] (Figure 1A). In addition to PLP, endocytic sorting and remodeling of the plasma membrane have also been reported for two other integral myelin proteins: MAG and Myelin-oligodendrocyte glycoprotein (MOG) [44]. Both MAG and MOG are localized to the myelin membranes and internalized by clathrin-dependent endocytosis. However, they are sorted differently with MAG sorted into LE/lys and MOG directed to recycling endosomes. On the other hand, PLP endocytosed by a cholesterol mediated and clathrin-independent pathway is directed to LE/lys until maturation [44]. During brain development, OPCs migrate from their site of origin to the axonal target, extending and wrapping their long processes around the axons and promoting the typical myelination process [45]. Adult OPCs expressing NG2 proteoglycan are known to divide and generate differentiating oligodendrocytes in adulthood [46]. Decreased colocalization of endocytosed NG2 with the lysosome was observed in conditional knockout OPC for Lgl1, a protein involved in cell polarity [47]. Additionally, it was revealed that in absence of Lgl1, NG2 escaped lysosomal degradation and rather recycled back to the plasma membrane. This provides evidence that in differentiating OPCs, Lgl1 acts as a positive regulator of NG2 trafficking to the lysosomes and its absence leads to attenuation of OPC differentiation due to abnormal NG2 recycling [47]. Lgl1 is also known to mediate lysosomal maturation as the lysosome of Lgl1-deficient OPC displays a tubular rather than a vesicular shape [47]. Furthermore, Lgl1 is reported to regulate vesicle acidification in the lysosomes [48], indicating a possible association between myelin proteins and lysosomal endocytosis.

### 2.3. Oligodendrocyte Autophagy and Energy Homeostasis

Autophagy plays a crucial role in the myelination process [49]. Specifically, in oligodendrocytes, autophagy helps in the removal of excess cytoplasm aiding in myelin compaction [50]. The mice harboring oligodendrocyte-specific mutation in Autophagy-related protein 5 (Atg-5), a key autophagy gene, showed severe tremor and survived only 12 postnatal days. Molecular analysis of the brain revealed apoptotic death of OPCs and significantly reduced differentiation and myelination in the Atg-5 mutants [50]. Additionally, increased expression of Microtubule-associated protein 1A/1B light chain 3 (LC3), an autophagosomal marker, was observed in the distal end of the oligodendrocytes. The myelin sheath made by Atg5-deficient oligodendrocytes was thicker than wild-type, due to the lack of autophagic clearance of cytoplasm [50], suggesting the use of autophagy as a potential therapeutic target to promote oligodendrocyte survival and repair of myelin post-injury. Autophagy inducers such as rapamycin act by inhibiting the mammalian target of rapamycin (mTOR), a protein that regulates energy metabolism by sensing nutrient availability and stress signals [51,52]. There is also evidence suggesting the role of mTOR in influencing oligodendrocyte differentiation [53]. The mTOR signals are activated during oligodendrocyte differentiation [53,54], whereas the inhibition of mTOR results in the arrest of oligodendrocyte differentiation at the late progenitor stage [53].

Abnormalities in myelination are often encountered in lysosomal storage diseases (LSDs) such as globoid cell leukodystrophy or Krabbe disease (KD), which is characterized primarily by the loss of myelin and accumulation of a cytotoxic metabolite, psychosine [55,56]. mTOR-independent autophagy enhancers, such as lithium, have been demonstrated to activate autophagy and improve cell viability post-psychosine exposure in an in vitro model of oligodendrocytes [57]. In another study, a fundamental autophagy marker p62 along with autophagosomes accumulated in the oligodendrocytes due to psychosine exposure [58], indicating a possibility that psychosine toxicity could be mediated via the autophagic/lysosomal pathway. The typical line of treatment for LSDs focuses on either increasing the activity of the specific target protein, alleviating enzyme defect, or modulation of lysosomal exocytosis [59,60]. Lysosomal exocytosis is mediated by lysosomal transcription factor EB (TFEB) that controls lysosomal biogenesis and autophagy [61]. In a genetic study using a zebrafish model, lysosomal G protein RagA was reported to be crucial for myelination by controlling the expression of TFEB. In RagA mutant oligodendrocytes, the expression of target genes of TFEB was upregulated, providing a piece of evidence that loss of TFEB function is needed for restoring myelination whereas hyperactive TFEB can result in decreased myelination [61]. Additionally, in a mouse model of focal demyelination, transcriptionally inactive TFEB was shown to increasingly colocalize in the cytoplasm of oligodendrocytes in promoting myelin growth [61]. Further study on the target genes and downstream signals of TFEB that control myelination may suggest potential therapeutic strategies for treating neurodegenerative demyelination.

## 3. Lysosomal Function in Astrocytes

Astrocytes are the most numerous cell type in the brain [62], and are a vastly physiologically heterogeneous population of cells (reviewed in [63]). They are involved in every aspect of CNS homeostasis, and subsequently, their lysosomal function is integral in maintaining a disease-free state in the CNS. In fact, inducing the astrocyte-specific deletion of sulfatase modifying factor 1, the gene associated with multiple sulfatase deficiency, a lysosomal storage disease, is sufficient to induce a neurodegenerative phenotype [64]. Therefore, proper lysosomal function in astrocytes is a necessary requisite for a healthy nervous system and subsequently systemic function. The information below details the known role astrocytic lysosomes have in releasing gliotransmitters, modulating plasma membrane plasticity, clearing neurodegenerative plaques and pruning synapses, and modulating the energy metabolism of astrocytes as well as neurons. Furthermore, we speculate on the future of enhancing astrocytic lysosomal function to target neurodegenerative diseases in a therapeutic fashion.

### 3.1. Astrocytic Exocytosis

Astrocytes have been dubbed gliocrine cells due to the secretion of numerous factors that influence the CNS. In fact, some astrocytes have a close physiological relationship with synapses forming a tripartite configuration enabling a bidirectional exchange of information between neurons and astrocytes. In the hippocampus, nearly 57% of synapses are in a close relationship with astrocytic endfeet [65]. The ability to respond to neural activity via the release of gliotransmitters has recently been implicated in a host of homeostatic functions to be discussed below; correlation has also been drawn between altered gliotransmission and the onset of neurodegenerative diseases [66,67]. Astrocytes of the tripartite synapse can sense neural activity by activation of cell surface G-protein coupled receptors (GPCRs) which induce cytosolic excitability triggering a spike in intracellular Ca^2+^ or cAMP [68,69]. Astrocytes secrete factors in a variety of means, including diffusion through transmembrane pores, transfer via plasma lamellar transporters and release by exocytic vesicles. Astrocytic secretory organelles include small clear vesicles, dense-core vesicles, secretory lysosomes, and multivesicular bodies. Secretory lysosomes are the largest of the vesicles, ranging from 300–500 nm in size, and are the primary astrocytic vesicle to undergo Ca^2+^ -mediated exocytosis [70].

Lysosomal exocytosis relies on the mobilization of internal Ca^2+^ ([Ca^2+^]_i_), which is necessary and sufficient to induce exocytosis. The flux of [Ca^2+^]_i_ can be achieved through a variety of external stimuli, most notably Ca^2+^, but also ATP, glutamate, hydrogen peroxide, ionomycin, mechanical stimulation, and UV flash photolysis [71,72,73,74]. Dissimilar to neuronal synaptic exocytosis, which occurs in less than a millisecond, astrocytic secretory lysosomes take orders of magnitude longer to be released. How the internal Ca^2+^ spike is elicited also plays a role in the kinetics and dynamics of secretion. Purinergic or metabotropic receptor stimulation causes oscillations in the levels of intracellular calcium and thus an extended period of release, with most fusion events occurring within the first 90 s; however, the period of release seems to extend nearly double that time. Stimulation with ionomycin or laser-induced injury elicits a mass fusion event with a majority of exocytotic events occurring within the first 20 s post-stimulation. These fusions taper out much more quickly compared to signaling receptor stimulation [75]. Moreover, differing pathways of induction correlate to differing modes of exocytosis. Receptor stimulation which induces local influxes of Ca^2+^ results in a partial fusion event, sometimes referred to as a “Kiss and Run”. It is worthwhile to note that this event allows the release of small and large proteins dissimilar to fibroblasts which have a similar mechanism with a restricted pore size wherein only small molecules are released. Additionally, plasma membrane injury in astrocytes can result in a massive influx of extracellular Ca^2+^, which triggers robust lysosomal exocytosis as a membrane repairing process [75].

The cargo of astrocytic secretory lysosomes not only hosts common lysosomal contents such as proteolytic enzymes, but have been shown to be implicit in the exocytosis of ATP. [71,72,74]. Secretory lysosomes host vesicular nucleotide transporter (VNUT)/solute carrier family 17 member 9 (SLC17A9). Using the ATP analog 2′/3′-O-(N-Methyl-anthroniloyl)-adenosine-5′-triphosphate (MANT-ATP), VNUT was identified as a transporter responsible for trafficking ATP into lysosomes [76]. It is evident that dense core vesicles (DCVs) also host ATP as cargo; however, more recent data have suggested that a majority of vesicular ATP is secreted by lysosomes [72,77]. Nonetheless, ATP release from astrocytes plays an important role in CNS homeostasis, regulating Ca^2+^ wave propagation [77], oxidative stress [73], and neuronal synaptic activity and thus plasticity [78] (Figure 1B).

Lysosomal exocytosis is also an important pathway for the targeting or repair of surface receptors on the plasma membrane [79,80]. Exposure to pro-inflammatory cytokines induces astrogliosis, resulting in morphological and molecular profile alterations. Astrocyte activation via TNF-α has been shown to induce surface expression of major histocompatibility complex II (MHC II) in a lysosomal exocytosis-dependent manner [79]. Astrocytic surface expression of MHC II has been implicated in the pathology and inflammation reaction of multiple sclerosis (MS) [80]. As mentioned above, in the event of membrane injury the influx of Ca^2+^ is immediate and robust eliciting complete fusion of the lysosome with the membrane, dumping all lysosomal contents extracellularly [75]. It is apparent that astrocytic lysosomes play a role in glia-transmission due to their role in releasing luminal contents in an inducible manner. The extent to which lysosomal release influences synaptic transmission is yet to be resolved. However, due to the close configuration of astrocytes and neurons in the tripartite synapse and the known functionality of ATP at the synapse, it is apparent that these cells and their lysosomes in particular play a role in the regulation of signal transduction in the CNS. Further elucidating the role of astrocytes in synaptic regulation and signal propagation could delineate mechanisms with therapeutic potential.

### 3.2. Astrocytic Phagocytosis/Endocytosis

In addition to responding to pathologic conditions, astrocytic phagocytosis is a routine activity performed for homeostatic functioning of the CNS. Innate astrocyte phagocytosis is instrumental in the development of neuronal circuits [81]. Astrocytes not only facilitate the formation and maturation of excitatory synapses through secretion of synaptogenic factors but are integral in the removal of redundant synapses that form during brain development. Direct astrocytic elimination of synapses is initiated in an activity dependent manner facilitated through the multiple Epidermal Growth Factor (EGF) like domains and tyrosine-protein kinase MER (MEGF10 and MERTK) phagocytic pathways which converge to the LE/Lys for subsequent degradation [82]. Moreover, astrocytes continue the pruning of synapses into adulthood contributing to the persisting plasticity of the brain. This has been recently been validated in the CA3-CA1 circuit in vivo, using an mCherry-eGFP reporter system that is used for monitoring autophagic flux [81]. The findings demonstrate the role of astrocytes in the elimination of excitatory synapses in a MEGF10 dependent manner. Furthermore, regulated endocytosis plays a role in maintaining membrane plasticity and composition in regions of interest. Aquaporin-4 (AQP-4) is a key regulator of water homeostasis in the CNS and localizes to the perivascular endfeet of astrocytes to regulate water flux through the blood–brain barrier. AQP-4 is co-expressed with the dystroglycan complex (DCG), a group of proteins that functions to interact with the laminin of the perivascular space thus confirming the localization of AQP-4. Moreover, laminin associated DCG preferentially interacts with inactive dynamin as opposed to active dynamin which would facilitate the recycling of AQP-4 complex to the endosome [83]. Another example of endocytosis regulating astrocyte surface composition is the cAMP-dependent endocytosis of glutamate transporters. Astrocytic glutamate transporters are localized to cellular niches in high densities such as the neuropil and regulate the extracellular concentration of glutamate, thus regulating the strength of excitatory synapse transmission. Therefore, an increase in cAMP concentrations facilitates the endocytosis of glutamate receptors and excitatory amino acid transporter 1/glutamate transporter-1 (EAAT1/GLT-1) [84].

Astrocyte phagocytic demand is greatly increased in the diseased CNS. Neurons are post-mitotic cells and thus protein aggregation in neurons cannot be dissipated via cell division. Therefore, the role of astrocytic clearance of pathogenic protein aggregates in neurodegenerative diseases is gaining more attention as an avenue for a therapeutic approach. Astrocytes have been implicated in the clearance of extracellular α-synuclein, amyloid-β, prion proteins, and Tau, [28,85,86,87] furthermore; there is evidence for the direct transfer of α-synuclein from neurons to astrocytes [88]. However, excessive phagocytosis of α-synuclein has been associated with incessant intercellular deposits, thus instigating mitochondrial stress by over-burdening the lysosomal degradation pathway [89]. Therefore, it has been hypothesized that upregulating astrocytic lysosomal number and function via pharmacological activation of lysosomal regulators such as TFEB and Sirtuin 1 (SIRT1) can facilitate increased cellular lysosome content and subsequent clearance of neurodegenerative associated proteins [90,91]. An instance of neuron to astrocyte metabolic coupling involves the transfer of toxic lipid droplets in an ApoE dependent manner. The resulting lipid particles are endocytosed by neighboring astrocytes and trafficked to the lysosome for degradation into useable fatty acid (FA) molecules which enter the β-oxidation cycle in the astrocytes [92], rescuing neurons from FA toxicity and repurposing the FA for energy production. Astrocytes also play a role in myelin clearance from lesions due to demyelinating pathologies such as MS, albeit a small role compared to microglia who are responsible for roughly 95% of the clearance. Myelin uptake was facilitated by the scavenger Lipoprotein receptor-related protein 1 and subsequently trafficked to the lysosome for degradation [93]. As mentioned before astrocytes are fundamental in shaping neuronal architecture in non-pathological conditions, this holds true in pathological conditions as well. In cases of middle cerebral artery occlusion, adjacent to the occlusion were reactive astrocytes which were positive for neuronal debris which was colocalized with the Lysosomal Associated Membrane Protein 2 (LAMP2). Indicative that the debris from degenerating neurons was processed by lysosomes [93]. A common characteristic of phagocytic astrocytes is the elevated expression of ATP Binding Cassette Subfamily A1 (ABCA1), which is also critical for preventing the ApoE mediated aggregation seen in AD [94,95].

It appears that the phagocytic capabilities of astrocytes are beginning to be realized as potential areas of therapeutic intervention. If the capabilities arise to elicit astrocyte phagocytosis independent of the toxic gain of function traits seen in astrogliosis the potential applications would be remarkable. The ability to reduce cellular debris, protein aggregates and decaying synapses would be a keen advancement to cope with neurodegenerative diseases and curb the excessive inflammation that is associated with many of them.

### 3.3. Astrocytic Autophagy and Energy Homeostasis

Autophagy in astrocytes is implicit in conserved autophagic functions such as stress response to nutrient starvation and cytosolic protein aggregation. Astrocytes play a role in the clearance of protein aggregates that are hallmarks of many neurodegenerative diseases. However, astrocytes are not immune to the effects of protein aggregation which has been implicated in the inhibition of autophagy and eventual toxicity leading to apoptosis in a model of PD [96]. Increasing levels of autophagic flux in astrocytes via rapamycin or progesterone have been shown effective in enhancing the neuroprotective and anti-inflammatory effect of astrocytes in models of PD and AD, respectively [97,98]. Alexander disease is a leukodystrophy caused by mutations and subsequent overexpression of a mutant allele of the glial fibrillary acidic protein (GFAP) gene; which is the major intermediate filament in astrocytes. The disease is associated with an increase in autophagic flux in an attempt to clear the GFAP aggregates; however, supplementing endogenous autophagic activation with the administration of rapamycin and/or lithium has been shown effective to induce further clearance of the mutant protein [99,100]. It is clear to see that in the CNS where many neurodegenerative diseases are caused by abnormal protein aggregation, its methodical elimination by autophagy would be crucial in maintaining homeostasis. Autophagy in astrocytes is further implicit in re-establishing homeostasis in instances of proteasomal inhibition, bilirubin induced cytotoxicity and oxidative stress [101,102,103]

Interestingly, astrocytes have been implicated as reservoirs for Human Immunodeficiency virus (HIV) in the human body [104]. Moreover, HIV-1 induces cellular alterations to promote replication and survival; inhibition of autophagy is one of these changes [105] via the HIV-1 associated protein Negative Regulatory Factor (Nef) [106], by disrupting the lipidation of LC3-I to LC3-II thus blocking the nucleation of the autophagosome. This inhibition of autophagy could be overcome by the administration of rapamycin, a potent mTOR inhibitor and inducer of autophagy [107]. The clearance of HIV from the CNS is imperative to curing the infection as astrocytic reservoirs have the potential to re-infect peripheral organs [108]. Akin to the implications associated with inducing astrocytic endocytosis, regulating autophagy in astrocytes may be an efficacious method of clearing cellular debris in neurodegenerative diseases.

## 4. Lysosomal Function in Microglia

Microglia are the resident phagocytotic immune cells of the CNS, representing 5–10% of total CNS cells. Microglia are the only neural cell of non-neuronal origin, originating in the yolk sac and invading the ectoderm at an early developmental time point that coincides with neuronal proliferation [109]. Although microglia are immune cells, the role microglia play in the CNS is much more than simple surveillance for and removal of pathogens [110]. Studies have shown that in healthy brain tissue, microglia have highly dynamic processes and constantly contact dendritic spines, axons, synapses, and other glial cells. Even more than their role in surveying the brain for pathogens and contacting neuronal processes, microglia have been implicated in sculpting synaptic connections and neurodegeneration [111,112,113]. The lysosome contributes to these functions, playing a role in the exocytosis of extracellular matrix proteases, endocytosis and phagocytosis of myelin debris, extracellular aggregates, and pathogens. Lysosomes are also critical regulators of microglial metabolism, allowing microglia to process a variety of energy substrates.

### 4.1. Microglial Exocytosis

Microglia modulate neuronal architecture via synaptic pruning and formation. Microglia were found to secret brain-derived neurotrophic factor (BDNF), a key growth factor involved in dendritic spine formation and neuronal survival [114] (Figure 1C). Secreted microglial BDNF regulates synaptic plasticity, by increasing the number of presynaptic terminals [115]. Neuronal architecture is also affected by the physical space in the synapse. For example, cathepsin S (CatS), a lysosomal cysteine protease exclusively expressed in microglia, degrades extracellular matrix (ECM) that eventually impacts the site of spine formation [116]. *CatS* knockout mouse model had significantly higher spine densities than wildtype controls [117]. However, the precise mechanism by which microglia release proteins by exocytosis is not fully understood. Moreover, multiple microglia-secreted proteins lack a signal sequence typically found on secreted proteins (reviewed in [118]).

In diseases, specifically proteinopathies, the role of the lysosomal exocytosis in microglia is more defined. In PD, buildup of α-synuclein occurs in neurons and microglia, and extracellularly [119]. One recent theory of PD pathogenesis implicates microglial phagocytosis of neuronal α-synuclein via lymphocyte-activation gene 3 (LAG3), which could spread the pathological aggregate of α-synuclein due to defective lysosomal degradation and exocytosis [120]. In AD, reactive microglia surrounding amyloid-β (Aβ) plaques and activation of inflammatory responses are prevalent. Aβ was found to increase microglial secretion of NF-κB, IL-1α, C1q, and TNF-α, as well as activating neuro-cytotoxic astrocytes, and thus creating a feedback loop of inflammatory activation [121]. Further research is required to understand if the modulation of this astrocyte-microglial inflammatory feedback loop could mediate a viable approach for intervention in AD. In lysosomal storage diseases, particularly sphingolipidoses, microglia accumulate undigested lipids impairing their ability to participate in myelin remodeling. Accumulated lipids block the endo-lysosomal pathway, causing microglia to secret pro-inflammatory cytokines, initiating a pathological inflammatory cascade [122]. This proinflammatory cascade has also been observed in demyelination, with myelin debris accumulating in microglia and triggering the formation of lipofuscin, an undegradable lysosomal aggregate of oxidized proteins and lipids [123].

### 4.2. Microglial Phagocytosis/Endocytosis

Microglia engulf pathogens and extracellular debris. They can do this via their ability to continuously survey the CNS [110]. Microglial engulfment of the neural synapse has been observed to occur during postnatal synaptic development, with recognizing synaptosome-associated protein 25 (SNAP25) and PSD95 (postsynaptic density protein 95), the markers of pre- and post-synaptic terminals, respectively [124]. Neuronal phagocytosis is controlled in a variety of ways. In adult hippocampal neurons, IL-33 was found to be expressed in an experience-dependent manner [125]. The IL-33 receptor, IL-1 Receptor-Like 1 (IL1RL1) is predominately expressed in microglia in the CNS, and ablation of IL-33 signaling decreased the number of dendritic spines. Furthermore, IL-33 upregulates extracellular matrix (ECM) turnover, by modulating the localization of Aggrecan in the lysosomes of microglia, and thus promoting spine elongation and maturation [116,126]. Microglia are the dominant producer of C1q protein in the complement pathway of the brain. The complement pathway increases microglial phagocytosis for neuronal synaptic pruning [124,127,128], and therefore is closely associated with the pathologic phagocytosis of synapses in the AD brain [127].

Microglia break down substrates that neurons are unable to process and excrete. Therefore, microglial dysfunction increases Aβ deposits along with upregulated CD68, a protein expressed in phagocytotic microglia. The Aβ deposits are also shown to increase in a sleep-deprived mouse model, suggesting a link between loss of sleep and lack of Aβ clearance [129]. This is further supported by the recruitment of microglia to Aβ deposits and the increase in phagocytosis of synapses during sleep [117,126,130].

Although microglia are thought of as the phagocytic cells of the CNS, increasing evidence is being brought forth that astrocytes are also active phagocytes, suggesting redundant functions of the glial cells. In fact, there is evidence that microglia and astrocytes communicate and coordinate phagocytic efforts in events of neuronal apoptosis [131]. Further studies on the signals and physical interaction between both cell types in the phagocytic pathway would be of great interest.

### 4.3. Microglial Autophagy and Energy Homeostasis

Autophagy in microglia is mainly studied in the context of senescence and disease, but also it plays a key role in healthy aging [132,133]. Autophagy serves as quality control on long-lived proteins and organelles and also as a way for the cell to mediate energy homeostasis [134]. Autophagy is regulated by various intracellular and extracellular sensors that function via the regulation of mTORC1, further reviewed in [135]. In the brain, increased autophagic flux is associated with protection from age-related neurologic deficits [136]. Age-related decline of autophagy is implicated in both AD and PD. Autophagic dysfunction has been shown to contribute to delays in remyelination after acute and chronic demyelinating insults [29,111,137,138]. In AD, microglia phagocytose and digest Aβ via the autophagic pathway. The process of autophagy of Aβ has been shown to impair autophagic flux and activate the nucleotide-binding oligomerization domain (NOD)-, leucine-rich repeats (LRR)- and pyrin domain-containing protein 3 (NLRP3) inflammasome, inducing neuronal damage [139]. This could prime a positive-feedback loop of neuronal apoptosis increasing the amount of extracellular Aβ that microglia are exposed to. Further, extracellular Aβ precedes neuronal degeneration and prompts a swift microglial response [130]. This suggests that early upregulation of microglial autophagy could slow the progression of AD and is a potential therapeutic target.

Normally, microglia uptake α-synuclein via LAG3 and degrade α-synuclein through LC3-positive autophagolysosomes [140]. However, in PD mutant cells, α-synuclein degradation does not occur because the autophagic flux is inhibited [137]. Dysfunctional lipid metabolism in the aged brain also contributes partially to α-synuclein aggregation. One lysosomal storage diseases, Gaucher disease, is caused by a mutation in the *GBA* gene that encodes glucocerebrosidase. This lysosomal hydrolase catabolizes lipid metabolites such as glucosylceramide and glucosylsphingosine. Using a human α-synuclein A30P transgenic mouse model crossed with the *GBA* mutant, Taguchi et al. found that an increased level of glucosylsphingosine is correlated with the extent of α-synuclein aggregation, and thus *GBA* dysfunction is closely associated with the increased risk for PD [141,142]. Whether the microglial lysosome initiates the pathologic insult or is a downstream amplifier of pathogenesis in neurodegenerative diseases has to be fully elucidated. Energy homeostasis is crucial for microglial function, as in pathologic conditions there may be a shift away from normally available energy substrates. Metabolic disruption could be triggered by ischemia, hypoglycemia triggered by improper diabetic management, genetic causes like Glucose transporter type 1 (GLUT1) deficiency, or hypoglycorrhachia (low cerebrospinal fluid (CSF) glucose) caused by bacterial meningitis [143]. Microglia shift metabolism via mTOR-dependent signaling, even in various metabolic states [144]. This ability to maintain a constant source of energy is critical for their role as immune cells. The ability to shift energy sources rapidly enables microglia to be responsive to CNS insults regardless of energy source.

## 5. Targeting Glial Lysosomal Pathways for Therapeutic Effects

Although research primarily focuses on the neuronal protein aggregates, recent studies have investigated the role of glial autophagic responses to proteinopathies. Glia have been shown to uptake and degrade protein aggregates in multiple proteinopathies, with astrocytes and microglia accumulating Aβ in AD [91,145], oligodendrocytes showing inclusions of α-synuclein in multiple system atrophy (MSA) [146], and astrocytes accumulating prion-associated protein in Creutzfeldt-Jakob disease [86].

With the increased awareness of glial degradation of CNS protein aggregates brings questions into the ability of therapeutic interventions to ameliorate or prevent disease. In an AD mouse model, microglial were found to upregulate autophagy to degrade Aβ fibrils, which also helped to regulate their NLRP3 inflammasome response, increasing neuronal survival [139]. Astrocytes have also been seen to activate the NLRP3 inflammasome in response to Aβ accumulation, an effect which was decreased upon treatment with rapamycin to induce autophagy. Increased astrocytic autophagy was also seen in vitro when treating cultured astrocytes with progesterone [147]. Sirtuin 1 (SIRT1) has been implicated in reducing the production of neuronal Aβ and has also been shown to upregulate lysosome number in astrocytes exposed to Aβ in culture [91]. These pathways could prove to be useful therapeutic targets to slow disease progression in AD.

Synucleinopathies are associated with impairment of the autophagy-lysosomal pathway. The importance of specifically targeting glial autophagy was recently highlighted in a model of MSA. In a study by Arotcarena et al., TFEB was upregulated in mouse models of PD and MSA [146]. In this study, broad expression of TFEB was shown to reduce α-synuclein toxicity in the PD model. Interestingly, in the MSA model, dopaminergic neuron-specific TFEB overexpression did not show any neuroprotection, whereas TFEB overexpression in oligodendrocytes was neuroprotective [146], indicating a critical role of oligodendroglial lysosomes. In a study investigating metformin administration in a 1-methyl-4-phenyl-1,2,3,6-tetrahydropyridine (MPTP) model of PD, it was found that metformin reduces dopaminergic neuron death and decreases α-synuclein accumulation [148]. Metformin also decreased the inflammatory response of microglia as well, decreasing the cytokine response typically seen in PD, though if this reduction is wholly beneficial has yet to be fully determined. Therefore, the glial autophagy-lysosomal pathway has a potential to be a key target in preventing and treating neurodegenerative disorders.

## 6. Concluding Remarks

It is evident that glial lysosomes play a key role in a variety of physiological functions. Oligodendrocytes use lysosomes to generate or remove myelin structure for plasticity. Astrocytes provide metabolites at the synapse via secretory lysosomal vesicles, contributing to the ability of the synapse to fire. Microglia remodel the extracellular matrix and process pathogenic material with their lysosomes. Further studies on the detailed molecular mechanisms on how lysosomes are generated and secreted by internal and external cues in each glial cell type, and how their malfunctions change the normal physiology of the cell, would provide insights into the development of novel therapeutics of neurodegenerative diseases that are closely associated with lysosomal dysfunction.

## Data Availability

Not applicable.
